# *Koji amazake* produced by double saccharification contains more isomaltose and modifies the gut microbiota in mice

**DOI:** 10.3389/fnut.2024.1489912

**Published:** 2024-11-06

**Authors:** Aito Murakami, Atsushi Saito, Fu Namai, Tadashi Fujii, Takumi Tochio, Jinichi Toida, Takeshi Shimosato

**Affiliations:** ^1^Graduate School of Medicine, Science and Technology, Shinshu University, Nagano, Japan; ^2^Food Technology Department, Nagano Prefecture General Industrial Technology Center, Nagano, Japan; ^3^Food and Feed Immunology Group, Laboratory of Animal Food Function, Graduate School of Agricultural Science, Tohoku University, Sendai, Japan; ^4^Department of Gastroenterology and Hepatology, Fujita Health University, Toyoake, Japan; ^5^Department of Medical Research on Prebiotics and Probiotics, Fujita Health University, Toyoake, Japan; ^6^BIOSIS Lab. Co., Ltd., Aichi, Japan; ^7^Institute for Aqua Regeneration, Shinshu University, Nagano, Japan; ^8^Department of Biomolecular Innovation, Institute for Biomedical Sciences, Shinshu University, Nagano, Japan

**Keywords:** koji amazake, Duncaniella, double saccharification, gut microbiota, mouse, Isomaltose, Muribaculum

## Abstract

*Koji amazake,* which is made from rice and rice *koji* (a product of *Aspergillus oryzae*), is a traditional Japanese beverage that has glucose as its main component. It also contains isomaltose, which has been reported to have various functionalities related to gut health. In the present study, we attempted to produce *amazake* with a higher concentration of isomaltose without using any additives by focusing on the saccharification step of rice *koji* production as a means of creating new value for *amazake.* Two types of rice *koji* that were obtained at different fermentation time points were used, and we changed the saccharification process from the usual one step of saccharification to two steps of saccharification using a different type of *rice koji* for each step. The *amazake* made by double saccharification (DSA) contained 20 times more isomaltose than the commercial *amazake* products. In an *in vivo* study, oral administration of the DSA modified the cecal microbiota in mice. Moreover, changes were seen in the abundances of several gut microorganisms, such as *Anaerotignum lactatifermentans, Muribaculum intestinale*, and *Parabacteroides merdae*. These findings indicate that our novel method may be useful for producing *amazake* with a high isomaltose content that may have health benefits in humans.

## 1 Introduction

*Washoku*, which means traditional Japanese cuisine, was registered in 2013 on the intangible cultural heritage list by the United Nations Educational, Scientific, and Cultural Organization. As the Japanese government has emphasized, the health benefits of *washoku* have been gaining much attention not only in Japan, but also around the world (1). *Washoku* often contains many and various fermented foods (2). Most Japanese fermented foods contain *koji*, which is a grain, such as rice, wheat, and/or soybeans, fermented with a non-pathogenic fungus, i.e.*, Aspergillus oryzae* or *Aspergillus luchuensis* (3); these fungi produce enzymes for the saccharification of the starch present in the grains (4).

*Amazake* is a drink that is one of the simplest traditional Japanese fermented products prepared using *koji*. There are two types of *amazake* that differ depending on the raw material used: *koji amazake* and sake-cake *amazake*. *Koji amazake* is made from rice and rice *koji* (*A. oryzae* products), and its manufacturing process consists of two main steps: preparation of the rice *koji* and saccharification. To make rice *koji*, brown rice is polished into white rice, gently washed in water, steeped, and steamed. The spores of *A. oryzae* are then inoculated onto the steamed rice. Next, the rice *koji* is mixed with water, and placed in a tank set at 50°C to 60°C for the saccharification (5).

The main component of *koji amazake* is glucose, which is produced from rice by breaking down rice starch; this is performed by an amylase secreted by *A. oryzae*. As a result, *koji amazake* mainly contains glucose rather than sucrose and fructose, which are found in many other beverages. Furthermore, various oligosaccharides are produced by transglycosylation, such as maltose (Glc(α1-4)Glc) and isomaltose (Glc(α1-6)Glc) (5). In particular, isomaltose has been gaining much attention as an alternative to sucrose due to its potential health benefits, such as its low glycemic index and slow hydrolysis (6–11), and probiotic potential (12, 13).

Accordingly, we believe that *amazake* with a high concentration of isomaltose may have more health benefits for humans and increase the value of *amazake* on the market. In a previous investigation to improve the quality of *koji amazake*, Oguro et al. (14) studied the relationships between temperature, saccharification, and oligosaccharide production, including isomaltose; they reported that modifications to the saccharification conditions resulted in only small changes to the concentration of isomaltose.

In this study, we focused on the rice *koji-*making process to find a way to increase the value of *amazake*, and established a method that significantly increased the isomaltose concentration in *amazake*. In the production of *amazake*, it is common to use one type of rice *koji* and to perform one saccharification step. However, in this study, we used two types of rice *koji* that were obtained at different fermentation time points, and saccharification was carried out in two steps using the two types of obtained rice *koji,* one for each saccharification step. The isomaltose concentration in the *amazake* produced using double saccharification (double-saccharification *amazake;* DSA) was increased as a result, which is a novel finding. Furthermore, administration of the DSA to mice induced changes in the gut microbiota. Here, we introduce our new two-step saccharification method for producing DSA, and report on the potential of DSA to confer health benefits, which is expected to increase the value of *amazake.*

## 2 Materials and methods

### 2.1 Starter *koji*

The starter *koji* used in this study (*koji* No. 1 and No. 2) were purchased from Akita Konno Co., Ltd. (Akita, Japan). *Koji* No. 1 is often used for making bean *koji*, and *koji* No. 2 is specifically used for making rice *koji* for sake. The starter *koji* strains were stored at 4°C until used for the experiments.

### 2.2 Preparation of rice *koji* and saccharification for making *amazake*

To examine the effects of different fungal strains on saccharification and the fermentation time, we separately used two types of starter *koji* (*koji* No. 1 and No. 2) to make rice *koji*. For making rice *koji*, we used *Akita Komachi* rice that was produced in 2023. The rice was gently washed in water, steeped overnight, and steamed for 1 h at normal pressure. Subsequently, the steamed rice was cooled to approximately 35°C, mixed with the *koji* (0.35 g/kg), and fermented in a bath maintained at 32°C during fermentation (for 42 h). At 18 and 25 h of fermentation, the rice *koji* samples were mixed by hand to break up the clumps that had formed and as a method to help decrease the temperature to the appropriate temperature range. The temperature of the rice *koji* was controlled during the fermentation process as follows: the temperature was 30°C at the start, then it was gradually increased to 38°C by 18 h, at which point the sample was mixed, then kept at 33°C until 25 h, at which point the sample was mixed again, and kept at 30°C until 42 h. The rice *koji* was sampled at 18, 25, and 42 h with the samples prepared using starter *koji* No. 1 referred to as samples X-1, Y-1, and Z-1, respectively, and those prepared using starter *koji* No. 2 referred to as samples X-2, Y-2, and Z-2, respectively. These samples were stored at-30°C until used in the experiments. For the experiments, 50 g of the obtained X, Y, or Z was mixed with an equal amount of water and incubated at 50°C for 6 h for the saccharification to make *amazake*.

### 2.3 Saccharification

For saccharification with the addition of maltose, 10 g of maltose was dissolved in 40 g of water. Subsequently, 40 g of each sample was mixed with the maltose solution and incubated at 50°C for 6 h, then sampled for the analysis of the sugar levels.

The DSA was prepared using the rice *koji* samples X-1 and Z-2 as follows: 50 g of sample X-1 was mixed with 50 g of water and incubated at 50°C for 6 or 24 h; subsequently, the obtained saccharification sample (60 g) was mixed with sample Z-2 (40 g) and incubated at 50°C for 6 or 30 h.

### 2.4 Analysis of the sugar concentrations in *amazake*

In addition to the DSA prepared in this study, commercial *amazake* products were also examined. For the glucose analysis, the *amazake* samples were diluted two-fold with distilled water and centrifuged at 1,870 *× g* for 10 min at 4°C. Aliquots of the obtained supernatants were diluted and analyzed for glucose using a glucose analyzer GA05 (A&T Corporation, Kanagawa, Tokyo). The amount of maltose or isomaltose in each sample was analyzed by high-performance liquid chromatography (HPLC) using a WATERS1525 (Nihon Waters K.K., Tokyo, Japan). In brief, 2 g of sample and 2 mL of 50% ethanol were mixed and sonicated for 30 min. After extracting the saccharides from each sample, the supernatant that was obtained by centrifugation at 1,870 *× g* for 10 min at 4°C was filtered through a 0.45-μm filter to remove the impurities. Then, each sample was analyzed by HPLC with a Shodex Asahipak NH2P-50 4E column (4.6 mm I.D. *×* 150 mm) at 35°C to quantify the maltose or isomaltose. The eluent was 70% acetonitrile, and the flow rate was set at 0.8 mL/min. The sugars in the samples were detected with a Refractive Index Detector.

### 2.5 Determination of the nutrient composition of DSA

Determination of the nutrient composition of the DSA was outsourced to LSI Medience Corporation (Tokyo, Japan). One-hundred grams of DSA was prepared as described above, and samples were sent to LSI Medience Corporation.

### 2.6 Mice and oral administration of the DSA

C57BL/6 mice (7 weeks old, female) were purchased from Japan SLC (Shizuoka, Japan). The mice were housed under controlled temperature and light conditions with *ad libitum* access to a standard diet (MF; Oriental Yeast Co., Ltd., Tokyo, Japan) and sterile water. The mice were acclimatized for a week, then randomly allocated into two experimental groups (N = 6 each): a phosphate-buffered saline (PBS) control group and a DSA group. Each mouse was orally administered PBS or a homogenate of DSA (200 μL/mouse) for 14 consecutive days (Days 1 to 14); the homogenate of DSA was prepared by centrifugation of the DSA at 1,400 *× g* for 5 min. On day 14, the mice were euthanized, and the cecal contents were collected.

### 2.7 16S rRNA gene sequencing

DNA was extracted from the cecal content samples using a fecal collection kit, and the 16S V3–V4 region of the DNA was amplified according to the methods of a previous report (15). The DNA was sequenced using the Illumina MiSeq platform (Illumina, San Diego, CA, USA) and a MiSeq Reagent Kit v3 (Illumina). Quantitative Insights into Microbial Ecology version 2 (Qiime2) bacterial flora analysis software (16) was used for microbiota analysis. Qiime2view was used to analyze the diversity and changes in the intestinal flora at the family and species levels based on the resulting qzv files. The *α*-diversity was analyzed using the observed features (amplicon sequence variants; ASVs) and Shannon diversity. The *β*-diversity was analyzed using the Jaccard and Bray-Curtis dissimilarities. In addition, multiple comparison tests using the Tukey–Kramer method were performed to analyze the intestinal bacteria that were significantly different between the DSA and PBS groups at the species level.

### 2.8 Statistical analysis

Statistical analysis was performed using Prism software (version 7; GraphPad Software, San Diego, CA, USA). Outliers were identified using the ROUT method (*Q* = 2%) and omitted before further statistical analysis. Statistically significant differences were assessed by two-tailed ordinary one-way analysis of variance followed by Tukey’s multiple comparisons test when statistical significance (*p* < 0.05) was indicated. Results are presented as the mean ± standard error (SE).

## 3 Results

### 3.1 The effects of double saccharification on the concentration of isomaltose in *amazake*

**Figure 1:**
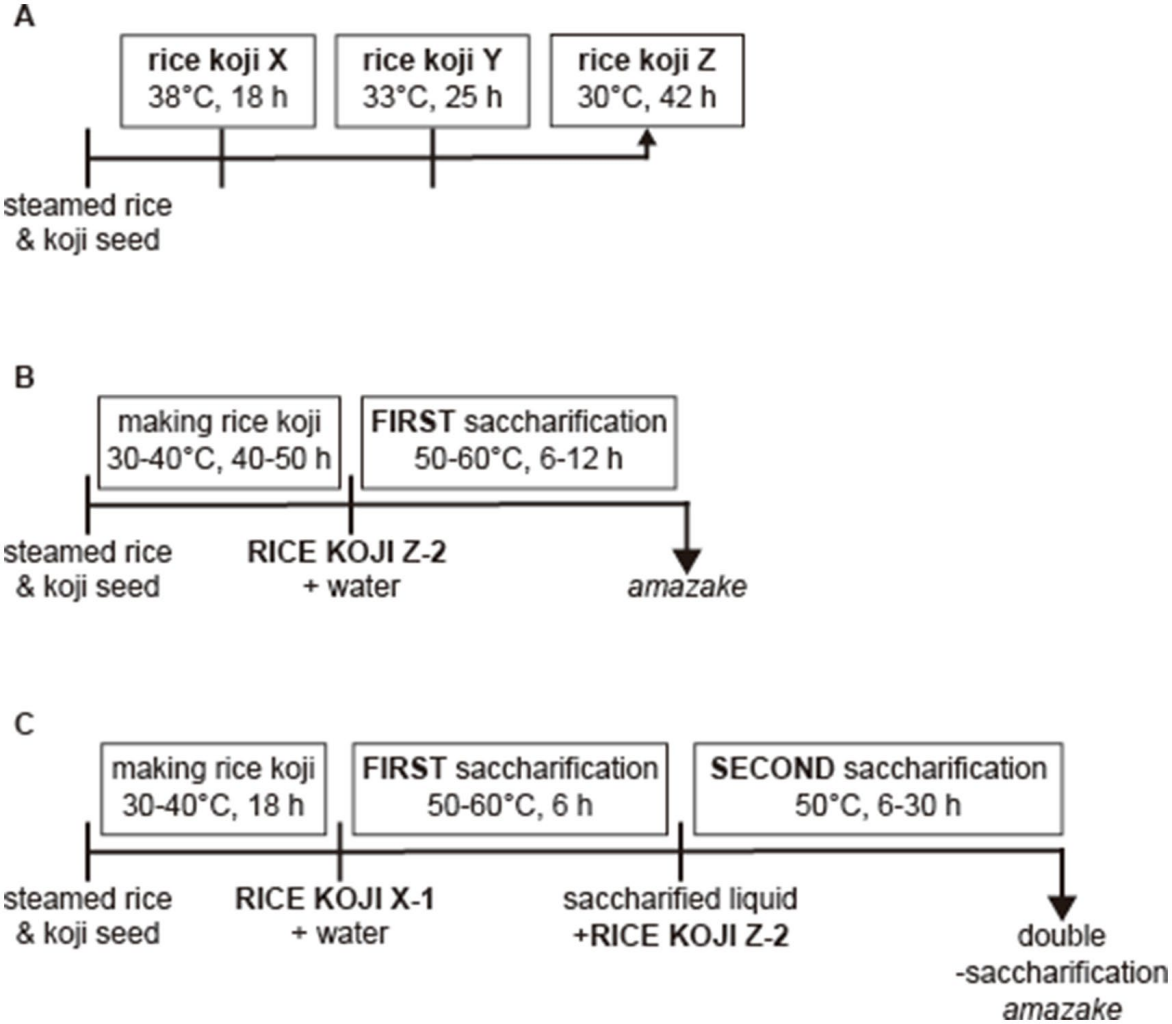
Scheme of the manufacturing of *amazake*. The schedule for rice *koji* sampling (A), the manufacturing steps for *amazake* (B), and for DSA (C).

**Figure 2:**
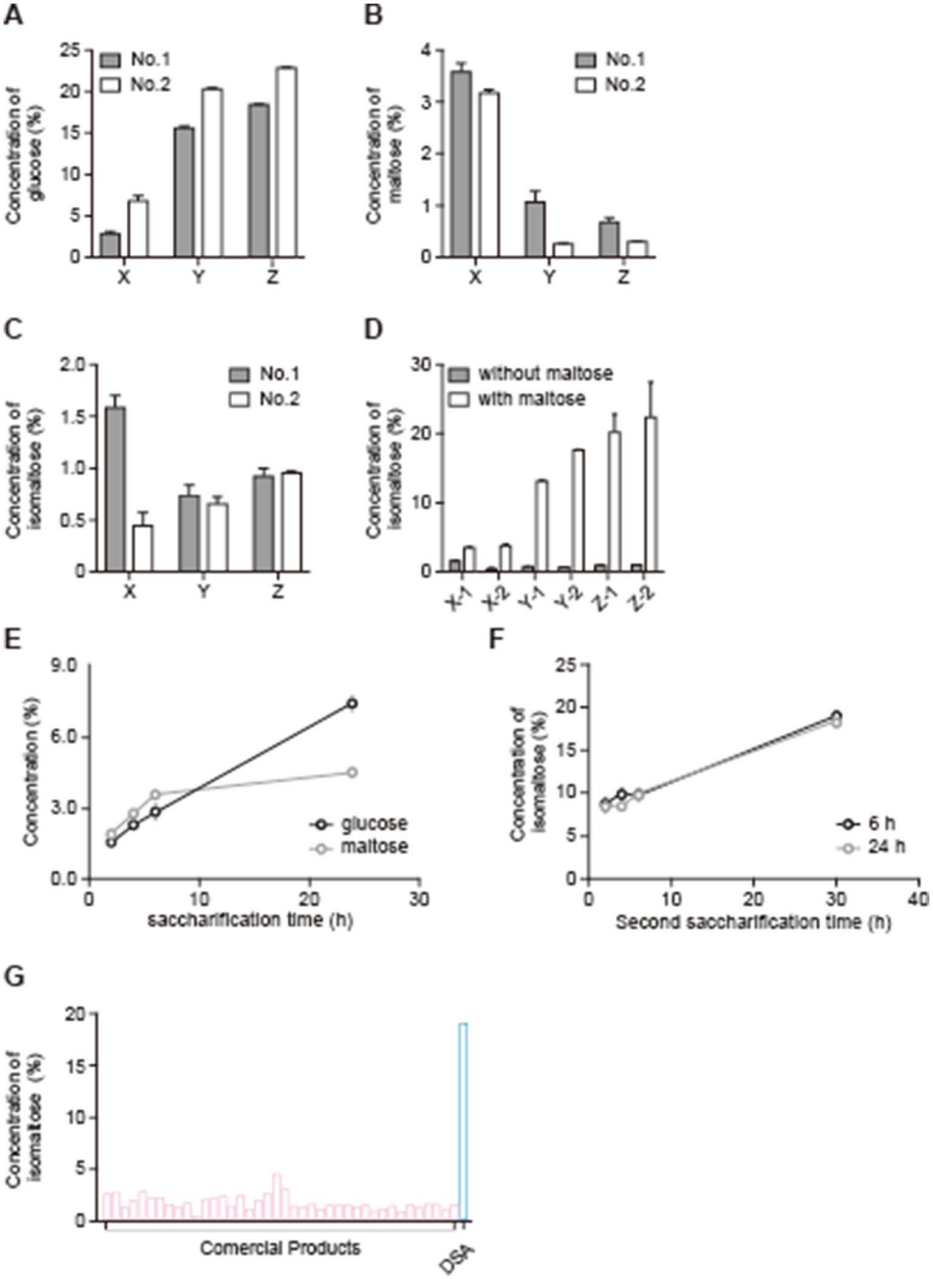
Variations in the concentration of each sugar due to the use of different starter *koji* and fermentation times, and increases in the isomaltose concentration in *amazake* due to double saccharification. Concentrations of glucose (A), maltose (B), and isomaltose (C) during *koji* production, concentrations of isomaltose during *koji* production with maltose addition (D), concentrations of glucose and maltose during the first fermentation (E), concentration of isomaltose during the second fermentation (F) using DSA, and comparison of isomaltose concentration in commercial *amazake* with that in DSA-produced *amazake* (G). Data are shown as the mean ± SE. *p < 0.05.

Figure 1 shows a scheme of the manufacturing steps for DSA. The nutritional information on the DSA is shown in Table 1. The rice *koji* was sampled at three different time points, i.e., at 18 h (samples X), 25 h (samples Y), and 42 h (samples Z) (Figure 1A). The concentrations of glucose, maltose, and isomaltose in each sample were measured to examine the relationship between the fermentation time and the concentration of each saccharide. The concentration of glucose was the lowest in samples X (18-h time point) regardless of the type of *koji* used (*koji* No. 1 or No. 2; Figure 2A). The concentration of maltose was highest in samples X (Figure 2B). The concentration of isomaltose did not differ significantly between the samples, but it was slightly higher in sample X-1 than in the other samples (Figure 2C). Considering that isomaltose is mostly formed from maltose through the transglycosylation activity of *α*-glucosidase, a saccharification test was performed with the addition of maltose to examine which sample had the highest transglycosylation activity. The concentration of isomaltose increased when maltose was added, and was highest in samples Z from the longest time point, especially in sample Z-2 (Figure 2D). To confirm which sample had the highest maltose content and to examine how the concentration of maltose depended on the time of saccharification, sample X-1 was saccharified for 24 h. The concentration of maltose increased until 6 h, and remained stable thereafter. In contrast, the concentration of glucose continued to increase until 24 h (Figure 2E). A second saccharification test was conducted to examine how the time of the first saccharification influenced the concentration of isomaltose in *amazake* samples produced using sample X-1 saccharified for 6 or 24 h. In both *amazake* samples, the level of isomaltose was approximately 20% at 30 h after the start of the second fermentation (Figure 2F). When the concentration of isomaltose in the DSA was compared to those in commercial products, we found that the DSA contained significantly more (almost 20 times more) isomaltose than the commercial products (Figure 2G).

**Table 1:** Nutritional information on the DSA.

General ingredients	Per 100 g
Energy	218 kcal
Moisture	46.00 g
Protein	3.50 g
Fat	0.50 g
Carbohydrates	49.90 g
Ash	0.10 g
Sodium	1.00 mg

### 3.2 DSA altered the diversity of the cecal microbiota

**Figure 3:**
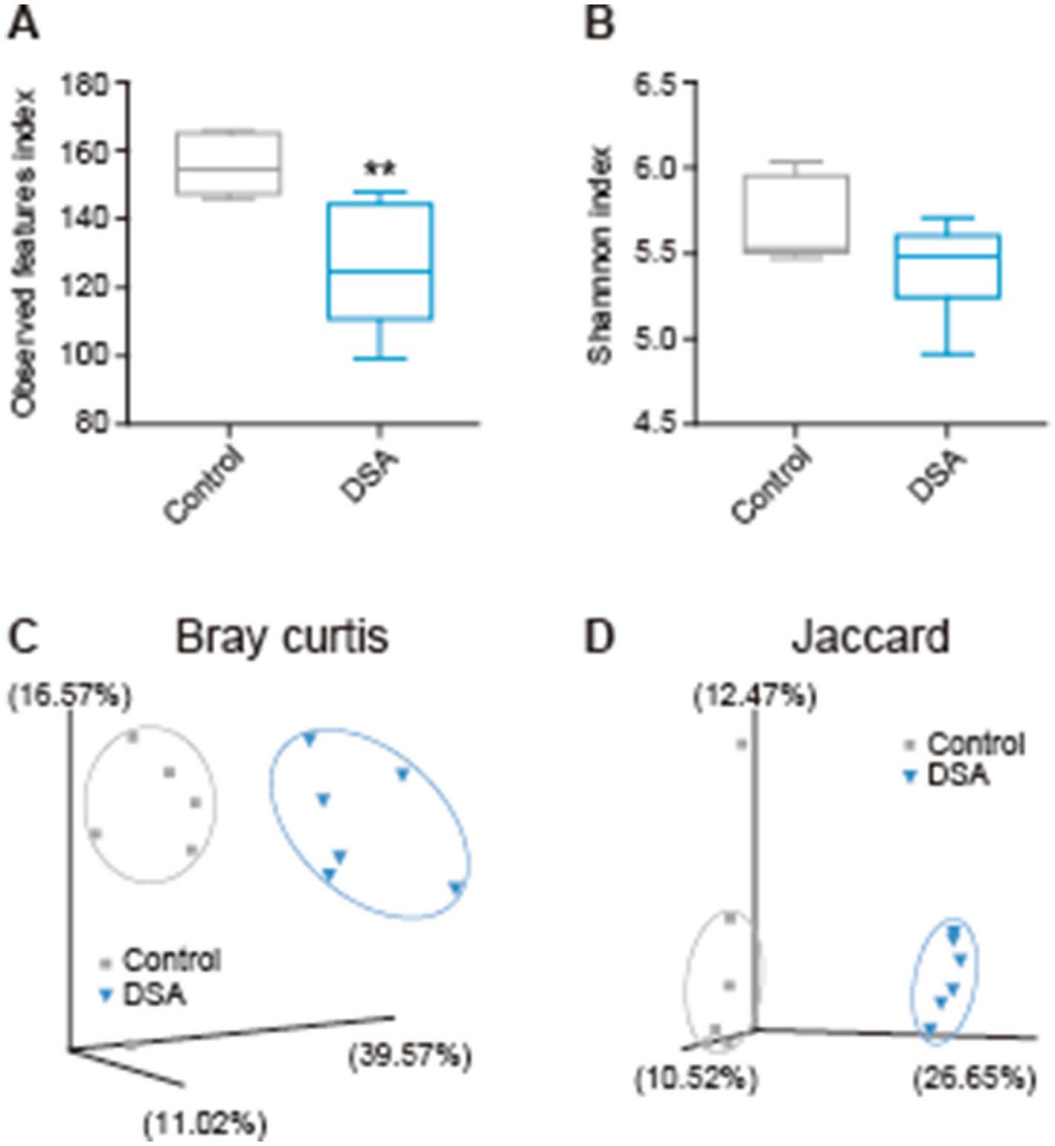
Variations in the diversity of the intestinal microbiota in mice administered DSA. Observed features index (A), Shannon index (B), and *β*-diversity analyzed using Bray-Curtis (C) and Jaccard (D) dissimilarities. *N* = 6. Data are shown as the mean ± SE. **p < 0.01.

**Figure 4:**
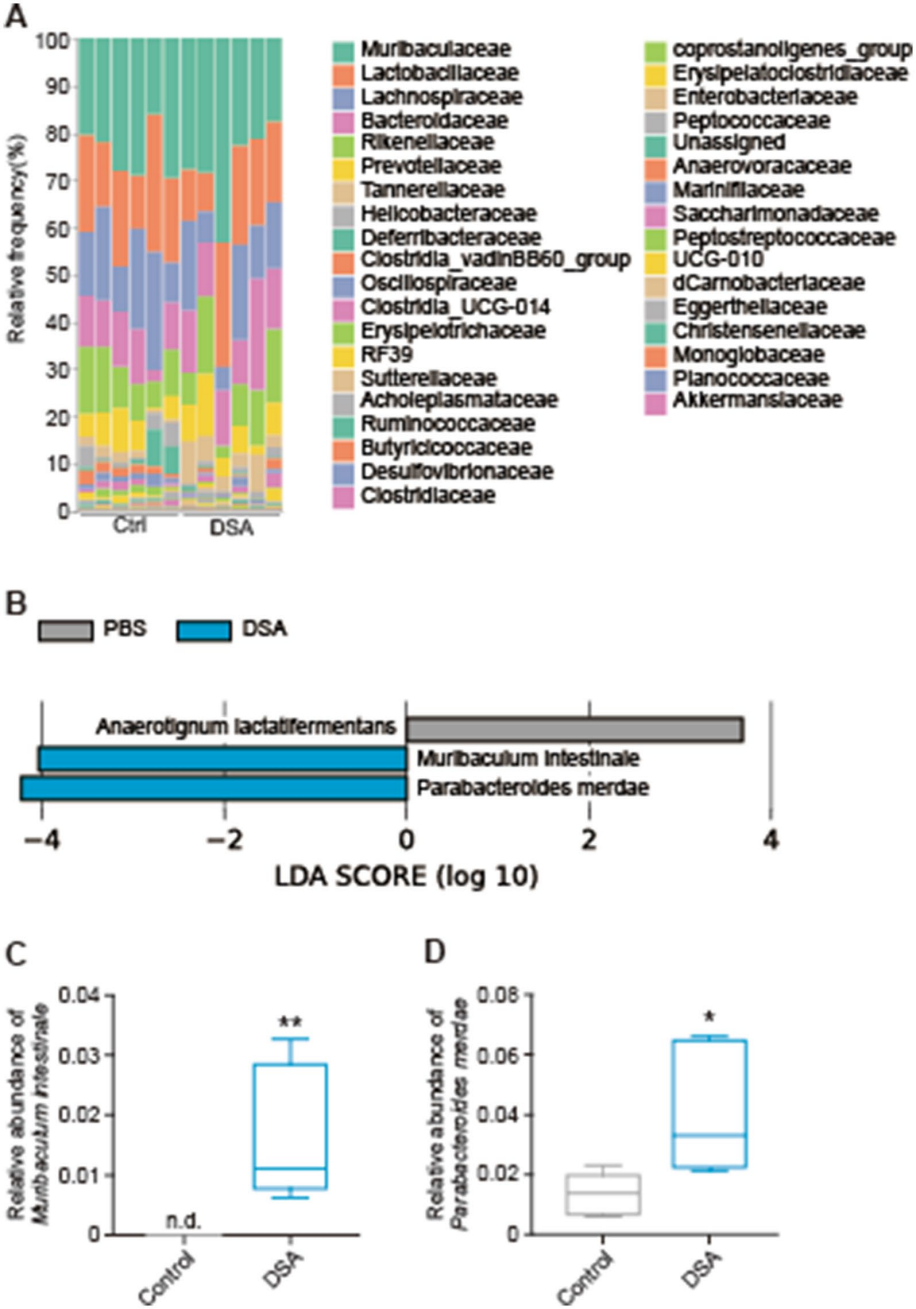
Composition of the gut microbiota and the relative abundances of several bacteria affected by DSA administration. A bar plot **(A)**, bacteria with significant differences in the ASVs at the species level **(B)**, the relative abundances of *M. intestinale* **(C)** and *P. merdae* **(D)***. N* = 6. Data are shown as the mean ± SE. *p < 0.05, **p < 0.01.

After 2 weeks of oral administration of PBS or DSA in mice, the cecal contents were collected and analyzed by next-generation sequencing, and the *α*-diversity was examined. There was a significant difference in the observed features between the PBS and DSA groups (Figure 3A), while the Shannon diversity index showed no significant increase in the α-diversity in the two groups (Figure 3B). Figures 3C,D show the Bray-Curtis and Jaccard *β*-diversity analysis results for the mouse cecal contents. The principal coordinate analysis results showed that the oral administration of DSA altered the gut microbiota (Figures 3C,D). The results of the taxonomic analysis are presented in Figure 4A as a bar plot (via QIIME2) showing the bacterial composition (at the family level) of the cecal content in each animal of each group. The significance of differences was investigated. The differences in the gut microbiota due to the administration of DSA were examined by linear discriminant analysis (LDA) at the species level from the LDA effect size (LEfSe) analysis at the species level. Three bacteria were extracted (Figure 4B), and a comparison of the relative abundances showed that the abundance of *Anaerotignum lactatifermentans* was decreased, and those of *Muribaculum intestinale*, and *Parabacteroides merdae* were increased in the DSA group when compared to the PBS group (Figures 4C,D).

## 4 Discussion

It is already known that the concentration of isomaltose in *amazake* is not affected by the temperature or the time of saccharification (14). Therefore, in this study, we focused on the rice *koji-*making process, which is an important step in manufacturing *amazake*, for increasing the concentration of isomaltose.

In the present study, we used a common rice *koji* production method: rice *koji* production was completed in 42 h with mixing to break up clumps at 18 h and 25 h, and the temperature conditions were strictly controlled. However, numerous other methods also exist, and they differ between sake or miso breweries and stores. In the present study, the rice *koji* obtained at 18, 25, and 42 h was used as rice *koji* samples X, Y, and Z, respectively, and the concentrations of glucose, maltose, and isomaltose were measured after 6 h of saccharification (Figures 2A–C). When we compared all samples, the glucose concentration was lowest in the sample from the earliest time point in the rice *koji* making process, which were samples X. However, the concentration of maltose was the highest in samples X. Glucose is the main component in *amazake*, and it is derived from rice starch. Rice starch is first broken down by the *α*-amylase and glucoamylase secreted by *koji* mold, resulting in maltose and glucose (5). The produced maltose is also broken down to glucose by the α-glucosidase secreted by *koji* mold. Therefore, the concentrations of these saccharides were affected by the degree of mycelial growth and the amounts of enzymes present. Indeed, samples X showed the lowest glucose level and the highest maltose level, likely because the activity of maltase, which degrades maltose, was low. In fact, the activity of *α*-glucosidase and maltase has been shown to increase with the fermentation time (17, 18); thus, it is likely that the activity of these enzymes was lower in samples X since the fermentation time was shorter than for samples Y and Z. However, we did not examine the enzyme activities in the present study, and they will need to be analyzed in the future to confirm this. In addition, the concentration of isomaltose was similar in all samples, although it was slightly higher in sample X-1. It is known that isomaltose is commonly synthesized from maltose and maltooligosaccharides through hydrolytic and transfer reactions by *α*-glucosidase with transglycosylation activity, especially when maltose is present at a high concentration (19). In other words, a low concentration of maltose in samples may cause the maltose to become glucose through a hydrolytic reaction.

According to these results and the fact that a high concentration of maltose is needed to produce isomaltose, we conducted a saccharification test with the addition of maltose. We found that the addition of maltose increased the concentration of isomaltose (Figure 2D). Moreover, it has been reported that the production of enzymes in *koji* increases with the fermentation time (17), which might explain why samples Z showed the highest concentration of isomaltose: samples Z likely contained the most *α*-glucosidase and possibly the highest transglycosylation activity.

In the previous investigation, it was revealed that a high concentration of maltose and the transglycosylation activity of α-glucosidase were necessary for increasing the content of isomaltose in *amazake*. Thus, we considered that it may be possible to increase the isomaltose content of *amazake* without the use of additives if two saccharification steps were conducted using sample X-1 for the first step and sample Z-2 for the second step (Figures 1, 2). Regardless of the first saccharification time (6 or 24 h), the effect of the second saccharification using sample Z-2 provided a significantly higher concentration of isomaltose when compared to that of the commercial *amazake* products. The reason no difference was seen between the 6 and 24 h time points in the first saccharification is likely because the concentration of maltose was similar in the two samples (Figures 2E,F).

In conclusion, we succeeded in finding a new method of *amazake* production for increasing the concentration of isomaltose without the use of additives. Our *amazake* contained approximately 20 times more isomaltose than the commercial products (Figure 2G). However, the concentration of essential enzymes, such as *α*-amylase, glucoamylase, and α-glucosidase, as well as their transglycosylation activities, were not checked when the two saccharification steps were performed. Also, in this study, sample X-1 was used for the first saccharification and sample Z-2 was used for the second saccharification, but the details of how the two kinds of *koji* samples interact remain unclear. Therefore, further experiments are needed in the future.

Since isomaltose is widely expected to be a prebiotic (30), we performed an *in vivo* experiment to test the effects of DSA administration in mice. No differences were seen in the Shannon index between the two groups. In addition, the *β*-diversity showed significantly different clusters. Although the DSA had little influence on the species diversity within a group, it modified the structure of the bacterial clusters in the DSA group when compared to the PBS group (Figure 3). Moreover, the abundances of three types of bacteria were found to have changed significantly due to the administration of DSA, namely*, A. lactatifermentans, M. intestinale*, and *P. merdae* (Figure 4). *M. intestinale* is highly associated with dietary habits, and there are some studies showing that the consumption of a western diet and cafeteria foods may decrease its abundance in the gut (20, 21). *M. intestinale* is a glycan-degrading and butyrate-producing bacterium, and it has been reported that the abundance of *M. intestinale* increased or recovered with improvements of obesity-and intestinal inflammatory disease-related factors that were affected by high-fat diets and dextran sulfate sodium administration (22, 23). Thus, the consumption of DSA may be useful and effective for promoting a stable and healthy intestinal environment. The abundance of *M. intestinale* may have increased in the gut of mice that were administered DSA due to the fact that it is a glycan-degrading bacterium and DSA is considered to have more sugar chains because it contains more isomaltose than the commercial products. On the other hand, *P. merdae* has been isolated from human feces, and has been reported to be associated with several diseases, such as hypertension, polycystic ovary syndrome, cancer, and obesity (24). For example, while *P. merdae was found in increased abundance in people* with hypertension (25) and polycystic ovary syndrome (26), an increase in the abundance of *P. merdae* in the gut has been reported to contribute to the treatment of obesity-related cardiovascular disease. This effect was due to the ability of *P. merdae* to catabolism branched-chain amino acids, mediated by the porA gene (27). In other words, DSA intake may contribute to the prevention of lifestyle-related obesity and related metabolic diseases by increasing the abundances of *M. intestinale* and *P. merdae* in the gut. Interestingly, not only has *P. merdae* been shown to be more abundant in the gut of those in the 90-to 99-year-old group in East China (28), but *Muribaculaceae* species, including *M. intestinale*, have also been reported to be associated with longevity (29). Thus, while we believe that habitual DSA consumption would confer continual health benefits, a more detailed understanding of the function of the bacterial strain altered by DSA administration requires further investigation.

In the present study, we introduced a novel method of producing *koji amazake* that contains a high concentration of isomaltose. We focused on the rice *koji*-making process instead of the saccharification step, and prepared two types of rice *koji* that were obtained at different time points and were subsequently used in two saccharification steps; this method resulted in an increased concentration of isomaltose. Furthermore, the administration of DSA increased the abundances of the obesity-and longevity-related organisms *P. merdae and M. intestinale,* which can degrade glycan. These results suggest that DSA consumption may confer health benefits and help maintain a healthy intestinal environment.

## Data Availability

The datasets presented in this study can be found in online repositories. The names of the repository/repositories and accession number(s) can be found in the article/supplementary material.
